# Remote Short Sessions of Heart Rate Variability Biofeedback Monitored With Wearable Technology: Open-Label Prospective Feasibility Study

**DOI:** 10.2196/55552

**Published:** 2024-04-25

**Authors:** Robert P Hirten, Matteo Danieletto, Kyle Landell, Micol Zweig, Eddye Golden, Renata Pyzik, Sparshdeep Kaur, Helena Chang, Drew Helmus, Bruce E Sands, Dennis Charney, Girish Nadkarni, Emilia Bagiella, Laurie Keefer, Zahi A Fayad

**Affiliations:** 1 The Dr Henry D Janowitz Division of Gastroenterology Icahn School of Medicine at Mount Sinai New York, NY United States; 2 Windreich Department of Artificial Intelligence and Human Health Icahn School of Medicine at Mount Sinai New York, NY United States; 3 The Hasso Plattner Institute for Digital Health at the Mount Sinai Icahn School of Medicine at Mount Sinai New York, NY United States; 4 The BioMedical Engineering and Imaging Institute Icahn School of Medicine at Mount Sinai New York, NY United States; 5 Center for Biostatistics Department of Population Health Science and Policy Icahn School of Medicine at Mount Sinai New York, NY United States; 6 Office of the Dean Icahn School of Medicine at Mount Sinai New York, NY United States; 7 Nash Family Department of Neuroscience Icahn School of Medicine at Mount Sinai New York, NY United States; 8 The Charles Bronfman Institute for Personalized Medicine Icahn School of Medicine at Mount Sinai New York, NY United States; 9 Department of Diagnostic, Molecular and Interventional Radiology Icahn School of Medicine at Mount Sinai New York, NY United States

**Keywords:** biofeedback, digital health, digital technology, health care worker, HCW, heart rate variability, mHealth, mobile health, mobile phone, remote monitoring, smartphone, wearable devices

## Abstract

**Background:**

Heart rate variability (HRV) biofeedback is often performed with structured education, laboratory-based assessments, and practice sessions. It has been shown to improve psychological and physiological function across populations. However, a means to remotely use and monitor this approach would allow for wider use of this technique. Advancements in wearable and digital technology present an opportunity for the widespread application of this approach.

**Objective:**

The primary aim of the study was to determine the feasibility of fully remote, self-administered short sessions of HRV-directed biofeedback in a diverse population of health care workers (HCWs). The secondary aim was to determine whether a fully remote, HRV-directed biofeedback intervention significantly alters longitudinal HRV over the intervention period, as monitored by wearable devices. The tertiary aim was to estimate the impact of this intervention on metrics of psychological well-being.

**Methods:**

To determine whether remotely implemented short sessions of HRV biofeedback can improve autonomic metrics and psychological well-being, we enrolled HCWs across 7 hospitals in New York City in the United States. They downloaded our study app, watched brief educational videos about HRV biofeedback, and used a well-studied HRV biofeedback program remotely through their smartphone. HRV biofeedback sessions were used for 5 minutes per day for 5 weeks. HCWs were then followed for 12 weeks after the intervention period. Psychological measures were obtained over the study period, and they wore an Apple Watch for at least 7 weeks to monitor the circadian features of HRV.

**Results:**

In total, 127 HCWs were enrolled in the study. Overall, only 21 (16.5%) were at least 50% compliant with the HRV biofeedback intervention, representing a small portion of the total sample. This demonstrates that this study design does not feasibly result in adequate rates of compliance with the intervention. Numerical improvement in psychological metrics was observed over the 17-week study period, although it did not reach statistical significance (all *P*>.05). Using a mixed effect cosinor model, the mean midline-estimating statistic of rhythm (MESOR) of the circadian pattern of the SD of the interbeat interval of normal sinus beats (SDNN), an HRV metric, was observed to increase over the first 4 weeks of the biofeedback intervention in HCWs who were at least 50% compliant.

**Conclusions:**

In conclusion, we found that using brief remote HRV biofeedback sessions and monitoring its physiological effect using wearable devices, in the manner that the study was conducted, was not feasible. This is considering the low compliance rates with the study intervention. We found that remote short sessions of HRV biofeedback demonstrate potential promise in improving autonomic nervous function and warrant further study. Wearable devices can monitor the physiological effects of psychological interventions.

## Introduction

### Overview

Mental health conditions are common, with approximately 25% of the population in the United States experiencing a mental health disorder in a given year [1]. Since the COVID-19 pandemic, there have been increasing rates of anxiety, depression, and other psychological conditions [2]. This has disproportionately impacted health care workers (HCWs) who are at a higher risk of depression, anxiety, insomnia, and distress compared to the general population [3-6]. Over half of the physicians and approximately 40% of the nurses in the United States experience burnout, almost twice that of other professions [7]. Additionally, during the COVID-19 pandemic, approximately 1 in 5 HCWs were experiencing some degree of posttraumatic stress disorder [8]. Thus, HCWs represent a vulnerable population in which further study of mental health interventions is needed.

Unfortunately, access to mental health services can be limited [9]. Digital technologies, including smartphone apps and wearable devices, provide an opportunity to improve health care access and aid mental health professionals in the management of psychological conditions. Collectively, they can assess subjective and objective metrics of psychological and physiological well-being. Apps can remotely collect validated psychological assessments while wearable devices are able to monitor physiological metrics such as heart rate variability (HRV), a hypothesized indirect measure of the autonomic nervous system (ANS) [10-13]. HRV is a measure of the physiological variation in the time intervals between adjacent heartbeats [14]. It is hypothesized to be generated by heart-brain interactions and ANS processes, reflecting the activity of the sympathetic and parasympathetic nervous system tone on heart rate [10,15].

Higher HRV has been associated with reduced frustration, higher performance, and positive psychological adjustments [16]. Reduced HRV has been associated with reduced self-regulation, variable degrees of psychological tension, and anxiety [17,18]. Oscillations in heart rate occur due to the influence of respiration on the sinoatrial node of the heart and central nervous system respiratory pacemaker fluctuations. Interestingly, at 1 resting respiratory rate, the relationship between breathing and heart rate is asynchronous, with the heart rate increasing following inhalation [19,20]. This respiratory sinus arrhythmia is controlled by the vagus nerve, with increased vagal output producing greater heart rate variation, thereby reflecting the parasympathetic influence on the heart [21]. It has been shown that the amplitude of HRV is related to breathing frequency with maximum effect at a breathing rate of 0.1 Hz or 6 breaths per minute [19].

Mind-body interventions, such as deep breathing exercises, can improve resilience, psychological well-being, physiological functions, autonomic imbalance, mood, cardiopulmonary output, and immune function [22-25]. Adaptive changes in the central nervous system, characterized as reduced sympathetic tone, have been described with these exercises [26-28]. Achieving deep breathing rates of 4.5-6.5 breaths per minute results in higher HRV indices compared to baseline, with higher parasympathetic and baroreflex function [29]. This has been shown to positively impact physical function; athletic performance; quality of life; and psychological features such as anxiety, depression, and resilience [30-32]. The individual breath per minute rate producing the optimal HRV effect (resonance frequency) can be determined from measures of the heart and respiratory rate in real-time biofeedback sessions [33]. Changes in HRV secondary to respiratory rate modification can create a positive feedback loop further increasing HRV respiratory changes, elicited through biofeedback [19].

Biofeedback is a self-regulatory behavioral method that trains individuals to control physiological function through real-time information about these physical parameters [34]. HRV biofeedback involves the real-time visualization of HRV metrics and breathing’s effect on this metric. It has been shown to increase HRV in adults [31,35-37]. There is significant empirical support for the use of office- or laboratory-based HRV biofeedback programs for the improvement of psychological conditions. In a recent meta-analysis of 14 studies, HRV biofeedback was shown to improve depressive symptoms in several psychophysiological conditions, as well as increase psychological well-being [38]. Large reductions in self-reported stress and anxiety have been demonstrated with HRV-directed biofeedback [31], as well as positive impacts on anger, athletic performance, sleep, and quality of life [39]. A systematic review of HRV biofeedback further demonstrated significantly improved symptoms of anxiety, depression, panic disorders, and posttraumatic stress disorder in 70% of the included studies [40].

However, despite the effectiveness of HRV-directed biofeedback, there are limitations to the implementation of such a technique. These interventions often rely on structured training and computer- or laboratory-based practice sessions that are often performed in the laboratory setting. This makes it challenging to broadly implement such techniques, limiting access to populations that may be most likely to benefit. This has prompted several studies using HRV biofeedback remotely and outside the laboratory setting with computer-based programs that demonstrated effect [36,41,42]. An additional significant obstacle to HRV biofeedback is the length of time required for each session, which can last up to 40 minutes [43]. Most also incorporate at least 1 laboratory session per week in addition to the daily home sessions [44]. These long and structured sessions, however, limit the ability of individuals to institute an HRV biofeedback program into their daily lives. Short sessions of HRV biofeedback might therefore provide a greater impact if they are able to elicit an autonomic response. Interestingly, short sessions of HRV biofeedback can successfully modify HRV and improve the regulation of emotional reactivity and therefore warrant further evaluation [45,46]. Gross et al [47] used 5 short 3- to 5-minute HRV biofeedback sessions. However, these were led by in-person practitioners. They demonstrated that HRV was successfully moderated and increased during these sessions; however, it was not changed overall from before training to after training. Deschodt-Arsac et al [45] furthered the evaluation of short-session HRV biofeedback by evaluating a twice-daily 5-minute biofeedback session in athletes, demonstrating an increase in autonomic function and a decrease in anxiety levels.

HRV measurements during and after biofeedback sessions evaluating physiological effects are often over brief periods and are in the clinic or laboratory setting. This limits the evaluation of its effectiveness on an individual’s physiological status and further restricts biofeedback sessions to the office setting. Wearable devices provide a potential means to assess HRV remotely, passively, and outside the laboratory setting and, thus, a possible means to monitor HRV biofeedback in a real-world setting. Wearable-based HRV assessment can be performed through either electrocardiography (ECG) or photoplethysmography (PPG). ECG is the gold standard for HRV assessment as the graphical representation of cardiac activity enables the calculation of beat-to-beat intervals with reliability to the millisecond level [48]. Most commercially available wearables and all wrist- or hand-worn devices that measure HRV rely on PPG technology. PPG tracks heartbeats by measuring the alterations of light from an LED that reaches a photodiode created by pressure changes in veins with each heartbeat [49]. Several studies have used wearable devices to assess response to HRV-directed biofeedback sessions. However, these have primarily used wearables that both monitor and implement biofeedback at the same time. Chung et al [50] demonstrated in a small pilot study that the Lief Smart Patch can assess and deliver HRV-directed biofeedback to effectively modify HRV. However, HRV assessments, generated from an ECG tracing, were over very brief periods around the biofeedback sessions. Similarly, Lin et al [51] demonstrated that using an HRV biofeedback wearable device for a least 4 weeks was needed to demonstrate an effect on HRV. However, studies that have evaluated sensor-type preference in biofeedback have found that participants prefer wrist- or arm-worn sensors for monitoring [52]. Given the ubiquitous use of commercial smartwatches, many of which measure HRV, there is an opportunity to expand HRV-directed biofeedback monitoring with such devices. Commercial devices such as the Apple Watch [12,53,54], fPolar V800 [13,55], Empatica E4 wristband [56], and Fitbit Charge HR [57] have been shown to generate valid and reliable assessments of heart rate and HRV, with high agreement with ECGs. Furthermore, the use of HRV calculated through PPG signatures has been shown to be a reliable and valid method for the assessment of HRV in the setting of HRV-directed biofeedback [58].

Thus, the potential benefits of short sessions of HRV biofeedback coupled with the growth of digital technologies and wearable devices present an opportunity to expand the application and monitoring of HRV-directed biofeedback. To evaluate this approach, we launched a feasibility study to evaluate smartphone-based short sessions of HRV biofeedback in HCWs and monitored its impact using common commercially available wearable devices.

### Objectives

The primary aim of the study was to determine the feasibility of fully remote, self-administered short sessions of HRV-directed biofeedback in a diverse population of HCWs. We hypothesized that fully remote HRV-directed biofeedback would have high compliance rates by HCWs. The secondary aim was to determine whether a fully remote, HRV-directed biofeedback intervention significantly alters longitudinal HRV over the intervention period. We hypothesized that HRV-directed biofeedback would significantly alter longitudinal HRV measurements. The tertiary aim was to estimate the impact of this intervention on metrics of psychological well-being. It was hypothesized that psychological well-being would improve with HRV-directed biofeedback. Study feasibility will be assessed by the percentage of HCWs who are at least 50% compliant with the intervention over the study period.

## Methods

### Ethical Considerations

This study has been approved by the institutional review board at the Icahn School of Medicine at Mount Sinai (STUDY-21-00596). The study was retrospectively registered on ClinicalTrials.gov (NCT05958329). All participants signed informed consent. All study procedures were performed in accordance with the ethical standards outlined in the Helsinki Declaration of 1975, as revised in 2000. The study data was deidentified, with each participants data linked to a unique study identification number. Additionally, all data was stored on Mount Sinai’s HIPAA compliant servers.

### Study Design

**Figure 1:**
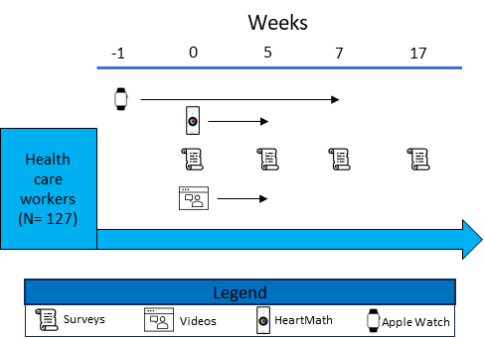
Participants were prospectively enrolled and followed for 17 weeks. Participants wore their Apple Watch for at least 7 days before starting the intervention period (week 0) and used it through week 7 of the study. The HeartMath device was used throughout the 5-week intervention period. Participants answered surveys at baseline, week 5, week 7, and week 17. In total, 5 weekly educational videos describing HeartMath and the basis behind the intervention were available for viewing through the 5-week intervention period.

The Warrior Shield study was an open-label prospective pilot clinical trial that enrolled HCWs across 7 hospitals in New York City (Figure 1). Participants were recruited from The Mount Sinai Hospital, Morningside Hospital, Mount Sinai West, Mount Sinai Beth Israel, Mount Sinai Queens, New York Eye and Ear Infirmary, and Mount Sinai Brooklyn. Eligible participants were aged 18 years or older, employees at 1 of the participating sites, had an iPhone series 6 or higher, and had or were willing to wear an Apple Watch 5 or greater. Potential participants were excluded if they had an underlying chronic disease or used a medication that is known to impact ANS function.

HCWs were recruited from the participating hospitals through emails sent to hospital employees and through study flyers (Figure S1 in Multimedia Appendix 1) placed in hospital common areas, including cafeterias and lobbies. Furthermore, participants who completed other digital studies run by our group were messaged with information about this study. Participants were provided with a US $50 gift card after completing 6 weeks of study activities. If a participant did not have an Apple Watch, he or she was able to borrow 1 for the duration of the study. This was returned to the research staff on completion of the study. Additionally, the participants had to return the HeartMath Inner Balance biofeedback device (HeartMath, LLC) at the end of the study.

### Study Procedures

#### Overview

Participants downloaded our ehive study app to their smartphones and self-verified inclusion and exclusion criteria before signing the electronic consent. Participants then electronically requested their HeartMath Inner Balance biofeedback device and an Apple Watch if they did not have 1 of their own wearable devices. Participants were recommended to wear the Apple Watch for a minimum of 8 hours per day. On receiving the Inner Balance device and after at least 7 days of wearing the Apple Watch, participants started the HRV biofeedback intervention, as described below. This continued for 5 weeks. Validated surveys to assess psychological well-being were completed at baseline in the ehive app. Surveys were repeated at week 5, week 7, and week 17. Participants were asked to wear the Apple Watch for at least 7 weeks after starting the intervention to enable HRV monitoring. They were reminded to participate in the study through regularly scheduled push notifications to their smartphones and automated email reminders sent by the study team.

#### ehive App

The ehive app is the centralized digital research platform of The Hasso Plattner Institute for Digital Health at Mount Sinai Hospital, New York, New York. The patient-facing portion of the platform is a smartphone app that enables electronic consenting of participants. Customizable patient-reported outcomes measures and other tasks such as the study surveys and weekly study videos are embedded in the app and can be tracked for compliance. This app can track participant compliance and engage participants through light touch measures such as customized push notifications and customized emails to participants to maintain engagement. It has been downloaded by over 1484 participants and has been used to collect over 51 million wearable-based data points and over 132,241 surveys [11].

#### Survey Instruments

Several validated surveys were evaluated throughout the intervention period. The 10-item Connor-Davidson Resilience Scale (CD-RISC 10) is a 10-question survey that measures resilience. Higher scores reflect higher resilience, with each question graded on a 5-point Likert scale [59]. The following question is an example of what is included in the survey: “I am able to adapt when changes occur.” The emotional support 2-item Patient-Reported Outcomes Measurement Information System (PROMIS) questionnaire is graded from 2 to 10 points, with higher scores reflecting higher perceived emotional support. It measures whether an individual has someone who will listen to them and with whom they can discuss their feelings [60]. An example of a question included in this survey is “I have someone who will listen to me when I need to talk.” The Perceived Stress Scale 10 (PSS-10) is a validated survey assessing perceived stress. It is 10 questions scored from 0 to 40, with higher scores correlating with elevated perceived stress [61]. An example item in this survey includes “In the last month, how often have you been upset because of something that happened unexpectedly?” The 2-Item Global Health and Quality of Life Scale asks participants to grade how their quality of life and health are in general. Higher scores correlate with lower health and quality of life [60]. The following is an example question included in this survey: “In general, would you say your health is excellent, very good, good, fair, or poor?” The Patient Health Questionnaire-4 (PHQ-4) is a 4-question survey that screens for anxiety and depression and is graded from 0 to 12 points. Higher scores reflect more severe impairment [62]. An example question from this survey is “Over the last week, how often have you been bothered by the following problem? Feeling nervous, anxious, or on edge.” The National Institutes of Health (NIH) PROMIS positive affect and well-being scale is a 23-question survey graded on a 5-point Likert scale. Higher scores reflect higher degrees of positive affect and well-being [63]. The following question is an example from this survey: “Lately I had a sense of well-being never, rarely, sometimes, often, or always?”

#### HeartMath Intervention

The HeartMath biofeedback system is developed by the HeartMath Institute, which is a nonprofit research and educational organization that develops and provides easy-to-use self-regulation tools focused on HRV biofeedback [64]. Its tools and techniques have been tested in a range of settings with good efficacy and uptake in conditions ranging from blood pressure, heart failure, stress, and trauma syndromes [65-68]. It is used in a range of settings and has been widely implemented in the health care industry, being offered to HCWs and patients at institutions such as Kaiser Permanente and the Veteran Administration Hospitals and Clinics [69,70]. The Inner Balance app combines a smartphone app (Figure S2 in Multimedia Appendix 1) with an optical ear sensor enabling real-time HRV visualization, assessment, and optimization during biofeedback sessions. Participants downloaded the Inner Balance app to their smartphones and set up an account using the login information provided by the study team. HeartMath’s Inner Balance pulse sensor clips on the participant’s ear and links through Bluetooth directly to an individual’s smartphone. The sensor contains an optical photodetector that samples up to 125 Hz providing real-time HRV assessment. Clip-on ear sensors have been shown to provide an accurate assessment of HRV compared to ECG [71]. Through HRV calculations, it produces an index of coherence, as a percentage of time in high, medium, or low coherence, through breathing and self-generated positive emotions [72]. A flower-shaped central visual pacer is present in the app, which paces a participant’s breathing. Through integration with sensed HRV, the app is able to reinforce the correct technique for HRV optimization [65,73,74].

Participants used the Inner Balance app for one 5-minute session per day for 5 weeks. Compliance was tracked remotely through the HeartMath system. HRV biofeedback sessions are usually supplemented with in-person or structured education sessions. To enable learning remotely, weekly educational videos were provided to participants in the custom ehive app. Five weekly videos provided information on (1) how to use the technology; (2) an introduction to HRV, biofeedback, and coherence; (3) a description of what coherence is and how it works; (4) how to incorporate biofeedback techniques into everyday life; and (5) reinforcement of what is learned in prior videos. Each video was less than 20 minutes in length and could be watched over the week.

#### Wearable Device

HRV was measured by the Apple Watch Series 5 or 6 that was worn by participants throughout the intervention and postintervention period. The Apple Watch contains a PPG optical sensor with both a green light diode and a light-sensitive photodiode [75]. This creates time series peaks that are filtered for ectopic beats and used to generate interbeat intervals. HRV was automatically calculated by the Apple Watch using the SD of the interbeat interval of normal sinus beats (SDNN) [76]. SDNN is a time-domain HRV metric that reflects both sympathetic and parasympathetic nervous system activity [10]. The only HRV metric available from the Apple Watch is SDNN. Multiple HRV measurements were generated by the Apple Watch throughout each 24-hour period in which individuals were wearing the device. These data were retrieved through our ehive app. The Apple watch calculates each of these SDNN measurements over 60-second windows, with a bias toward nighttime measurements, to minimize artifacts in the readings. The algorithms used by the Apple Watch for artifact rejection and ectopic beat handling are proprietary and not publicly available. However, they likely use well-described algorithms in this space [64]. While this is a limitation, the PPG-based HRV calculations from Apple Watches have been validated against ECG [12,77].

### Statistical Analysis

Data are presented descriptively as the mean and SD or frequency and percentages, as appropriate. Mean values for each psychological assessment were obtained at baseline; just before initiation of HRV biofeedback; and at week 5, week 7, and week 17. Changes over time in the psychological assessment were analyzed using mixed effects models with participants as random effects. Week-5, week-7, and week-17 survey results were each compared for statistical differences to the baseline values.

HRV is captured by the Apple Watch in a relatively sparse and nonuniform sampling and follows a circadian pattern [78,79]. To account for frequent daily measures of HRV that are collected from wearable devices over a several-week period, statistical methods that take into account these changes are needed. Daily circadian rhythms have been previously modeled by nonlinear cosinor methods [80]. This approach models the circadian HRV rhythm each day over 24 hours and enables the data to be described using circadian parameters (Figure S3 in Multimedia Appendix 1): (1) midline-estimating statistic of rhythm (MESOR): the midline of the rhythm, or a rhythm-adjusted mean, over the 24 hours; (2) acrophase: a measure of the time of the highest values that reoccur each day; and (3) amplitude: characterizes half the extent of the variation in every 24 hours. To fully use the cyclical nature of the physiological metrics, as well as the longitudinal measurements, mixed-effect cosinor models were used to model HRV over time based on the *cosinoRmixedeffects* R package (R Core Team) [81]. This expands the nonlinear cosinor methods to account for correlations of repeated measurements within a participant over time. As has been previously described, a cosinor model uses the nonlinear function Y(t)=M + *Acos*(2*π*t/τ + *ϕ*) + e_i_(t), where τ is the period (τ=24 h), M is the MESOR, A is the amplitude, and ϕ is the acrophase. This can be converted into the linear model with x=sin(2*π*t/τ), z=sin(2*π*t/τ). HRV can be written as Y(t)=M + *β*x_t_ + γz_t_ + e_i_(t) [82]. The mixed-effect cosinor model extends the linear framework in a longitudinal setting through the inclusion of random effects θ_i_ that models the within-patient correlation and expressed as Y_it_ = (M + *β*x_it_ + γz_it_) + W_it_θ_i_ + e_i_(t), where θ_i_ ~ MVN(0, Σ) [81]. Bootstrapping procedures were used to calculate the confidence intervals of the model estimates. Age, sex, and BMI were included as covariates in the HRV analyses with participants as random effects.

HRV was evaluated using the above approach for each 7 days of the study. The baseline measurement reflects the 7 days preceding the initiation of the HRV-directed biofeedback. Each subsequent 7-day period, over the 7-week HRV observation period, was compared to this baseline value. All analyses were carried out at the 2-sided .05 significance level using SAS (version 9.4; SAS Institute Inc) and R (version 4.2.2; Foundation for Statistical Computing). Since this was a proof-of-concept study, there was no adjustment for the multiplicity of hypothesis testing.

## Results

### Quantitative Findings

In total, 127 participants consented to the study between July 2021 and April 2022. The mean age of these participants was 37.3 (SD 10.6) years, with 93 (73.8%) being female. In total, 72 (56.7%) of these participants started the intervention and used the Inner Balance device at least 1 time (>0% compliance), while 49 (38.6%) participants were at least 20% compliant, and 21 (16.5%) participants were at least 50% adherent over the 5-week intervention period (Table 1).

**Table 1:** Demographic information for participants signing consent, those who used the intervention at least 1 time, those with at least 20% compliance, and those with at least 50% compliance.

Characteristics		Signed consent (N=127)	Compliance >0% (n=72)	Compliance ≥20% (n=49)	Compliance ≥50% (n=21)
Age (years), mean (SD)		37.3 (10.6)	38.4 (11.0)	38.0 (11.0)	37.7 (12.1)
Male, n (%)		33 (26.2)	22 (31)	13 (26.5)	5 (23.8)
BMI (kg/m^2^), mean (SD)		25.3 (5.5)	25.3 (5.5)	25.3 (5.6)	27.3 (6.5)
**Race, n (%)**					
	Asian	32 (25.6)	15 (21.4)	11 (22.9)	2 (10)
	Black	15 (12)	9 (12.9)	6 (12.5)	3 (15)
	White	69 (55.2)	43 (61.4)	29 (60.4)	14 (70)
	Native Hawaiian or Pacific Islander	2 (1.6)	1(1.4)	1 (2.1)	1 (5)
	Unknown	4 (3.2)	2 (2.9)	1 (2.1)	1 (5)
Hispanic or Latino, n (%)		29 (23.8)	16 (23.5)	11 (23.4)	6 (30)
Smoking: never or rarely, n (%)		103 (81.1)	57 (79.2)	40 (81.6)	17 (81)
Anxiety, n (%)		29 (24.4)	16 (23.5)	10 (20.4)	6 (28.6)
Depression, n (%)		25 (19.8)	15 (20.8)	10 (20.4)	6 (28.6)

The percentage of participants who watched the entire weekly video decreased throughout the study. A video introducing the study at enrollment was watched in its entirety by 100% of participants. The video in week 2 was watched in its entirety by 54% (68/127) of participants, the week 3 video was watched by 47% (60/127) of participants, the week 4 video was watched by 42% (53/127) of participants, and the week 5 video was watched by 39% (49/127) of participants. There was a technology tutorial video that provided information about the Inner Balance system. This was watched in its entirety by 65% (83/127) of participants.

Overall, the acceptability of the study was good. Participants were asked how satisfied they were with the HeartMath Intervention on a scale of 1 (not satisfied) to 7 (very satisfied). In total, 79 participants answered the question with a median score of 5. Out of the 81 participants who answered the question as to whether they pursued additional learning about HeartMath outside of the study, 17.3% (n=14) reported in the affirmative. Participants who pursued additional learning about HeartMath were more satisfied with the HeartMath intervention, scoring their degree of satisfaction with a mean of 6.07 (SD 0.86) compared to those who did not pursue outside learning (mean 4.45, SD 1.52).

### Psychological Assessment

In participants who were at least 50% compliant (n=21) with the Inner Balance device, resilience scores were noted to numerically increase between the baseline assessment, week 5, week 7, and week 17. However, none of these values differed significantly from the baseline assessment. Social support scores (emotional support 2-item PROMIS questionnaire) similarly demonstrated a numerical increase from baseline (mean 8.13, SD 1.46) to week 5 (mean 8.60, SD 0.89), week 7 (mean 9.80, SD 0.45), and week 17 (mean 8.67, SD 2.31). None of these increases were statistically significant compared to the baseline assessment. Stress scores (PSS-10) numerically decreased in the ≥50% compliant cohort, dropping from mean 20.63 (SD 5.95) at baseline to mean 10.67 (SD 7.77) at week 17. The change in stress scores at week 5 (*P*=.24), week 7 (*P*=.45), and week 17 (*P*=.26) were not significantly different compared to the baseline assessment. PHQ-4 scores, which increase when there is greater psychological impairment, decreased from baseline through week 17. In the ≥50% compliant cohort there was not a statistically significant change in these values, compared to the baseline assessment, at week 5 (*P*=.83), week 7 (*P*=.55), or week 17 (*P*=.38). NIH PROMIS positive affect and well-being scores rose as well over the 17 weeks in the ≥50% compliant cohort, reflecting increasing positive affect and well-being. Due to the small number of individuals in this cohort, we were not able to calculate *P* values for this comparison. The 2-Item Global Health and Quality of Life Scale increased over the observation period, demonstrating higher quality of life. This change, compared to baseline, did not reach the level of statistical significance at week 5 (*P*=.50), week 7 (*P*=.62), or week 17 (*P*=.36). Overall, psychological assessments additionally demonstrated numerical improvement over the 5-week intervention period and through the 17-week follow-up period in those who used the Inner Balance device at least once and in those who were ≥20% compliant, though they did not differ significantly from the baseline assessments (Table 2).

**Table 2:** The mean psychological assessments are presented at baseline, week 5, week 7, and week 17 in each compliance group. The mean scores for each survey at week 5, week 7, and week 17 are compared against the baseline scores. *P* values reflect the significance of this comparison. Compliance groups are defined as those performing the intervention at least 1 time, those with at least 20% compliance, and those with at least 50% compliance.

Measures			Compliance >0%				Compliance ≥20%				Compliance ≥50%		
			Mean (SD)		*P* value		Mean (SD)		*P* value		Mean (SD)		*P* value
**CD-RISC 10^a^**													
	Baseline	27.05 (7.20)		—^b^		28.04 (7.80)		—		27.38 (5.66)		—	
	Week 5	27.67 (6.80)		.15		27.31 (5.92)		.79		23.80 (7.66)		.68	
	Week 7	27.94 (9.16)		.05		28.22 (9.00)		.12		30.40 (6.95)		.44	
	Week 17	32.50 (3.42)		.07		31.33 (3.06)		.37		29.00 (4.58)		.95	
**Emotional support 2-item PROMIS^c^ questionnaire**													
	Baseline	8.45 (1.75)		—		8.48 (1.86)		—		8.13 (1.46)		—	
	Week 5	8.72 (1.13)		.41		8.77 (1.09)		.95		8.60 (0.89)		.65	
	Week 7	9.00 (2.0)		.12		9.00 (2.00)		.27		9.80 (0.45)		.27	
	Week 17	9.5 (1.0)		.70		9.33 (1.15)		.43		8.67 (2.31)		.66	
**PSS-10^d^**													
	Baseline	16.53 (6.22)		—		17.26 (6.31)		—		20.63 (5.95)		—	
	Week 5	18.61 (6.41)		.22		16.31 (5.34)		.64		17.00 (4.64)		.24	
	Week 7	18.82 (6.59)		.20		18.22 (6.24)		.53		16.80 (7.85)		.45	
	Week 17	12.63 (7.91)		.85		10.17 (7.59)		.23		10.67 (7.77)		.26	
**PHQ-4^e^**													
	Baseline	3.13 (2.53)		—		3.61 (2.59)		—		4.25 (2.92)		—	
	Week 5	2.61 (2.38)		.27		2.69 (2.53)		.19		3.60 (3.78)		.83	
	Week 7	2.71 (2.39)		.97		2.44 (1.01)		.40		2.60 (1.14)		.55	
	Week 17	0.88 (0.63)		.12		0.83 (0.76)		.08		1.33 (1.53)		.38	
**NIH^f^ PROMIS positive affect and well-being scale**													
	Baseline	82.65 (18.08)		—		84.91 (18.00)		—		81.50 (10.88)		—	
	Week 5	86.16 (13.14)		.23		85.21 (11.89)		.99		83.80 (15.59)		N/A^g^	
	Week 7	88.99 (16.55)		.19		90.33 (20.37)		.32		93.60 (19.48)		N/A	
	Week 17	97.50 (10.66)		.10		97.33 (13.05)		.18		90.00 (4.24)		N/A	
**2-Item Global Health and Quality of Life Scale**													
	Baseline	7.58 (1.54)		—		7.43 (1.44)		—		7.63 (1.41)		—	
	Week 5	7.61 (1.72)		.78		7.31 (1.84)		.60		8.60 (1.34)		.50	
	Week 7	7.41 (0.80)		.70		7.33 (1.00)		.84		7.20 (0.84)		.62	
	Week 17	7.88 (1.44)		.63		8.50 (0.87)		.19		8.67 (0.58)		.36	

^a^CD-RISC 10: Connor-Davidson Resilience Scale.

^b^Not available.

^c^PROMIS: Patient-Reported Outcomes Measurement Information System.

^d^PSS-10: Perceived Stress Scale 10.

^e^PHQ-4: Patient Health Questionnaire-4.

^f^NIH: National Institutes of Health.

^g^N/A: not applicable.

### Physiological Metrics

**Figure 2:**
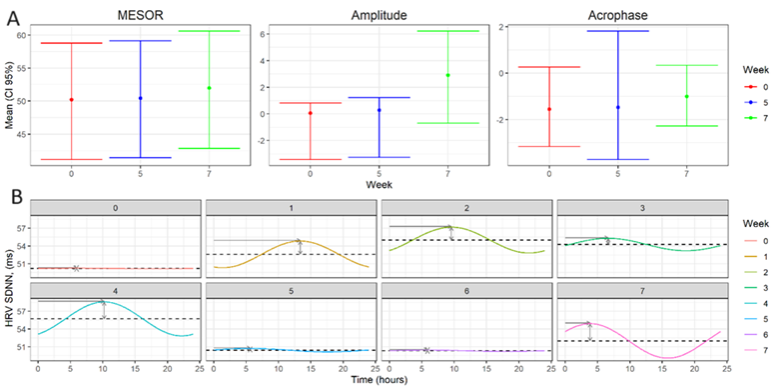
(A) Plots show the mean (95% CIs) heart rate variability (HRV) midline-estimating statistic of rhythm, amplitude, and acrophase for participants at baseline, week 5, and week 7. (B) Plots show the average weekly circadian HRV rhythm for participants at baseline and over the first 7 weeks of the study period for participants with at least 50% compliance (n=21). midline-estimating statistic of rhythm; SDNN: SD of the interbeat interval of normal sinus beats.

There was an average of 4.7 (SD 3.5) HRV measurements obtained per participant per day. The average length of time of each sample was 59 seconds. The median SDNN value obtained in the full cohort was 38 milliseconds with a minimum and maximum value of 10 milliseconds and 200 milliseconds, respectively. We fit a cosinor model evaluating differences in HRV (SDNN) each week over the 5-week intervention period and over the 2 weeks following the intervention period. There were no significant changes from baseline in the amplitude or acrophase of the circadian pattern of SDNN in all 3 compliance groups (Table 3). Significant changes were noted in the MESOR of the circadian pattern of SDNN in participants who are ≥50% compliant with the intervention. In this group, the mean MESOR was 50.20 (95% CI 41.16-58.78) during the baseline 7-day period. A numerical but not significant rise (*P*=.12) in the MESOR was observed during week 1 of the intervention (mean 52.59; 95% CI 43.65-61.08). There were significant changes in the mean MESOR of the circadian pattern of SDNN found during week 2 (mean 55.00; 95% CI 46.11-63.37; difference 4.80; 95% CI 1.63-7.91; *P*<.001), week 3 (mean 54.25; 95% CI 45.27-62.76; difference 4.04; 95% CI 0.64-7.00; *P*=.01), and week 4 (mean 55.70; 95% CI 46.77-63.94; difference 5.50; 95% CI 2.31-8.60; *P*<.001) compared to baseline (Figure 2). The MESOR during week 5 of the intervention and the 2 weeks after the end of the intervention did not demonstrate significant changes compared to baseline.

In the participants who used the Inner Balance device at least once and in those who were ≥20% compliant with the intervention, there was only 1 significant change in the MESOR observed over the 7-week follow-up period. There was a significant change in the MESOR of the circadian HRV pattern in participants with >0% compliance with the intervention during week 1 (mean 45.46; 95% CI 39.30-51.58; difference 1.48; 95% CI 0.10-2.88; *P*=.04), compared to the baseline 7-day period.

**Table 3:** The mean midline-estimating statistic of rhythm (MESOR), amplitude, and acrophase are presented for each week of the observation period. heart rate variability (HRV) circadian parameters were calculated for each 7 days of the study, with the baseline readings representing the 7-day preintervention period. Comparisons between each HRV metric 7-day period and the baseline 7-day period were performed. P-values reflect the significance of each comparison. Compliance groups are defined as those performing the intervention at least 1 time, those with at least 20% compliance, and those with at least 50% compliance.

Parameters			Compliance >0%							Compliance ≥20%							Compliance ≥50%				
			Mean (95% CI)		Difference (95% CI)		*P* values		Mean (95% CI)			Difference (95% CI)		*P* values		Mean (95% CI)			Difference (95% CI)		*P* values
**MESOR**																					
	Baseline	43.98 (37.84 to 49.93)		—^a^		—		45.71 (38.60 to 52.93)			—		—		50.20 (41.16 to 58.78)			—		—	
	Week 1	45.46 (39.30 to 51.58)		1.48 (0.10 to 2.88)		.04		46.83 (39.65 to 54.11)			1.12 (–0.53 to 2.97)		.19		52.59 (43.65 to 61.08)			2.38 (–0.84 to 5.14)		.12	
	Week 2	45.30 (39.46 to 51.70)		1.62 (0.13 to 3.31)		.05		46.93 (39.75 to 54.20)			1.22 (–0.68 to 2.88)		.18		55.00 (46.11 to 63.37)			4.80 (1.63 to 7.91)		<.001	
	Week 3	44.83 (38.80 to 50.85)		0.85 (–0.60 to 2.50)		.26		47.19 (40.19 to 54.36)			1.48 (–0.35 to 3.30)		.10		54.25 (45.27 to 62.76)			4.04 (0.64 to 7.00)		.01	
	Week 4	45.28 (39.08 to 51.42)		1.30 (–0.17 to 2.74)		.10		47.24 (40.03 to 54.51)			1.53 (–0.33 to 3.17)		.10		55.70 (46.77 to 63.94)			5.50 (2.31 to 8.60)		<.001	
	Week 5	45.31 (39.22 to 51.43)		1.33 (–0.14 to 2.95)		.09		47.25 (40.08 to 54.52)			1.54 (–0.17 to 3.46)		.09		50.43 (41.43 to 59.14)			0.22 (–3.47 to 3.69)		.89	
	Week 6	45.36 (39.23 to 51.42)		1.38 (–0.13 to 2.88)		.08		47.20 (40.07 to 54.36)			1.49 (–0.22 to 3.26)		.11		50.33 (41.41 to 58.84)			0.12 (–2.82 to 2.95)		.93	
	Week 7	45.19 (39.17 to 51.22)		1.22 (–0.44 to 2.75)		.14		46.66 (39.53 to 53.82)			0.95 (–0.87 to 2.66)		.31		52.00 (42.84 to 60.60)			1.79 (–1.46 to 4.90)		.27	
**Amplitude**																					
	Baseline	4.30 (2.61 to 5.94)		—		—		3.55 (1.57 to 5.61)			—		—		0.06 (–3.43 to 0.82)			—		—	
	Week 1	4.37 (2.63 to 6.15)		0.07 (–2.08 to 2.10)		.94		4.45 (2.49 to 6.33)			0.90 (–1.23 to 3.12)		.43		2.25 (–1.17 to 5.22)			2.20 (–2.52 to 4.98)		.25	
	Week 2	4.83 (3.12 to 6.58)		0.53 (–1.76 to 2.58)		.63		4.75 (2.88 to 6.62)			1.20 (–1.06 to 3.62)		.28		2.18 (– 0.99 to 5.03)			2.13 (–2.63 to 5.20)		.27	
	Week 3	4.45 (2.59 to 6.18)		0.15 (–2.02 to 2.34)		.88		3.75 (1.80 to 5.71)			0.20 (–2.35 to 2.58)		.88		1.05 (– 2.20 to 2.87)			0.99 (–2.87 to 3.70)		.55	
	Week 4	4.49 (2.69 to 6.23)		0.19 (–2.18 to 2.01)		.87		4.73 (2.63 to 6.79)			1.18 (–1.31 to 3.99)		.34		2.87 (– 0.62 to 6.16)			2.81 (–2.23 to 5.65)		.17	
	Week 5	3.91 (2.03 to 5.67)		–0.39 (– 2.56 to 1.75)		.71		3.67 (1.55 to 5.68)			0.12 (–2.01 to 2.66)		.92		0.29 (– 3.27 to 1.23)			0.23 (–2.98 to 3.32)		.89	
	Week 6	4.28 (2.56 to 5.96)		–.0.02 (– 2.26 to 1.95)		.98		4.08 (2.16 to 5.95)			0.53 (–2.00 to 3.12)		.64		0.12 (– 3.54 to 1.06)			0.06 (–3.04 to 3.45)		.96	
	Week 7	2.84 (0.99 to 4.60)		–1.46 (– 3.50 to 0.69)		.16		2.10 (0.08 to 4.16)			–1.45 (– 3.73 to 0.91)		.21		2.91 (– 0.69 to 6.23)			2.85 (–2.21 to 5.61)		.15	
**Acrophase**																					
	Baseline	–2.93 (–3.35 to –2.53)		—		—		–3.03 (–3.65 to –2.33)			—		—		–1.56 (–3.16 to 0.27)			—		—	
	Week 1	–2.79 (–3.19 to –2.40)		0.14 (–0.44 to 0.61)		.59		–3.25 (–3.71 to –2.81)			–0.22 (–0.93 to 0.49)		.49		–3.50 (–5.35 to –1.70)			–1.94 (–4.31 to 1.18)		.11	
	Week 2	–2.69 (–3.05 to –2.37)		0.24 (–0.24 to 0.75)		.29		–3.23 (–3.69 to 2.79)			–0.19 (–0.82 to 0.42)		.53		–2.50 (–4.17 to –0.50)			–0.95 (–3.32 to 1.24)		.26	
	Week 3	–2.56 (–2.88 to –2.24)		0.37 (–0.07 to 0.93)		.15		–2.47 (–2.89 to –2.05)			0.56 (–0.04 to 1.30)		.10		–1.74 (–2.33 to –1.08)			–0.18 (–1.35 to 2.03)		.70	
	Week 4	–3.32 (–3.72 to –2.92)		–0.39 (–0.96 to 0.14)		.16		–3.37 (–3.86 to –2.91)			–0.34 (–1.07 to 0.39)		.32		–2.67 (–4.10 to –1.01)			–1.12 (–3.35 to 1.33)		.20	
	Week 5	–3.16 (–3.61 to –2.72)		–0.23 (–0.84 to 0.33)		.41		–3.16 (–3.81 to –2.54)			–0.13 (–0.91 to 0.66)		.70		–1.47 (–3.72 to 1.81)			0.09 (–4.60 to 2.00)		.90	
	Week 6	–2.78 (–3.19 to –2.37)		0.15 (–0.27 to 0.69)		.54		–3.03 (–3.56 to –2.50)			0.0004 (–0.79 to 0.69)		>.99		–1.54 (–3.26 to 0.56)			0.02 (–3.26 to 1.55)		.98	
	Week 7	–2.89 (–3.50 to –2.20)		0.04 (–0.67 to 0.68)		.91		–2.87 (–4.04 to –1.67)			0.16 (–0.93 to 1.33)		.73		–1.01 (–2.27 to 0.34)			0.55 (–1.39 to 2.77)		.32	

^a^Not available.

## Discussion

### Overview

Overall, we found that using brief remote HRV biofeedback sessions and monitoring its physiological effect using wearable devices, in the manner that the study was conducted, was not feasible. This is considering the low compliance rates with the study intervention. However, there was a numerical improvement in all psychological metrics over the intervention period, and compliant participants had a measurable physiological change in wearable assessed HRV. In addition, participants were in general satisfied with the HeartMath system that was used. This supports the potential for at-home, HRV-directed biofeedback and wearable-based monitoring to be effective, but only when participants are engaged. The findings highlight the challenges with maintaining engagement in large remote intervention studies.

This study built on the existing literature supporting the use of short sessions of HRV biofeedback by using a short 5-minute HRV biofeedback session that could be performed on an individual’s smartphone. Furthermore, it took the structured education that often accompanies biofeedback and divided it into easily digestible short videos, which individuals could absorb at their own pace. This framework pilots an approach that enables the intervention to be used by individuals who might not have the time to engage in a more structured program. Furthermore, while the physiological effects of biofeedback are often evaluated through brief HRV assessments, we used a commonly used commercial wearable device to monitor its impact. While HRV data were available from the HeartMath device during the short biofeedback sessions, this represented only a very brief assessment of the physiological effect in a relatively small number of compliant participants (n=21). These measurements do not assess the intervention’s sustained effect on an individual’s physiological parameters, which is of primary interest in this study. Therefore, our focus was on analyzing and leveraging the longitudinal HRV data provided by the Apple Watch. The benefit of this approach is 2-fold in that it can unobtrusively monitor the intervention’s effect and evaluate the intervention’s impact over longer periods through its assessment of circadian features of autonomic function. Importantly, we demonstrated that the MESOR of the circadian pattern, which reflects the mean HRV reading over the observation period, increased in participants compliant with the intervention, reflecting increased parasympathetic tone. Previous studies have demonstrated that commercially available wearable devices may be able to monitor and identify psychological states through HRV monitoring [82,83]. The results of this study extend these observations by demonstrating that commonly used wearable devices can potentially be used to monitor the physiological effects of psychological interventions and warrant further evaluation.

We used the HeartMath system, which uses a well-studied HRV biofeedback tool, as described above. We found that psychological metrics were numerically improved with the intervention. However, these changes did not meet statistical significance. A primary driver of this observation is likely the low rate of adherence, as the number of people who were at least 50% compliant with the intervention was only 21 individuals. While the trend in improvement was evident in all 3 adherence groups, statistical significance may have been met if the number of participants ≥50% compliant was larger or if rates of adherence were well over 50%. Given the limited number of participants, we were not able to perform a sensitivity analysis to determine the minimum adherence or engagement rate needed to elicit an effect. However, the trends we observed in psychological metrics warrant further study of this approach. Another potential hypothesis as to why we did not see statistical improvement in the psychological metrics may be that the cohort is relatively healthy compared to other groups undergoing psychological interventions. However, when we look at psychological metrics such as resilience, we see that the mean CD-RISC 10 score for the entire cohort was 27.05, compared to the general population’s mean of 31.8 (SD 5.4) [84]. Therefore, the cohort is less resilient at baseline and presumably would benefit from such an intervention. Interestingly, we did demonstrate that short sessions of HRV biofeedback are able to significantly modify HRV. The performance of just half or more of the 5-minute biofeedback sessions in 1 week significantly impacted the circadian features of HRV and increased parasympathetic tone. While we did not observe this significant difference during the fifth week of the intervention in this cohort, the sample size was small, and engagement varied week by week, likely explaining the drop in effectiveness in the final week. During the 2 weeks after the intervention period, HRV was not significantly different from baseline. This observation demonstrates that sustained employment of short sessions of biofeedback is required for ongoing physiological effects. This is an important finding, as there are scarce studies evaluating the long-term impact of HRV biofeedback on HRV metrics, with few studies demonstrating sustained short-term effects [85,86]. Further studies evaluating the duration of physiological effects are needed.

While 127 participants initially joined the study, only 21 (16.5%) participants used the intervention at least half of the time. While low rates of persistent engagement can be seen across remote digital psychological intervention studies, future work using this remote biofeedback intervention should focus on direct means to maintain engagement. This could include coaching models or community-based engagement such as “leaderboards.” [87] Additionally, dedicated study coordinators checking in with each participant could potentially improve adherence and participant engagement. Adherence may be increased by focusing recruitment efforts on individuals most interested in biofeedback programs. Our recruitment methods opened the study up to any HCW across multiple hospitals. However, focusing recruitment efforts on individuals interacting with hospital psychological support systems would engage individuals more likely to be interested in performing psychological interventions. Furthermore, we could hypothesize that the most engaged participants may have some degree of knowledge or interest in digital technologies, given the employment of apps and wearable devices. Therefore, such programs may be most effectively deployed in tech-savvy populations. While 79 participants rated the acceptability of the Heart Math system and were overall satisfied with it, we, unfortunately, did not have qualitative data regarding its acceptability or feasibility.

There are several additional limitations to this study. One important limitation is the limited external evaluation of Apple Watch–generated HRV measurements. There have been several studies evaluating and validating the Apple Watch’s accuracy in measuring HRV. These studies have compared calculations derived from metrics collected from the Apple Watch with ECG measures. Turki et al [54] demonstrated that in 6 healthy participants, HRV acquired from R-R interval estimates derived from Apple Watch measures of heart rate are reasonable estimates of HRV derived from an ECG. Similarly, Hernando et al [12] validated the R-R intervals derived from the Apple Watch and the HRV metrics calculated from these series against readings derived from a single lead ECG acquired from the Polar band in 20 participants. Khushhal et al [53] performed a similar study on 21 individuals during exercise, demonstrating agreement in HRV metrics calculated from Apple Watch outputs compared to the Polar HR monitor. These studies demonstrate that Apple Watch metrics, used in the calculation of HRV by the device, are valid. However, the algorithms describing how the Apple Watch cleans the PPG data for ectopic beats and artifacts are not publicly available, and therefore limited data demonstrating how Apple calculated HRV metrics compared to ECG-derived measures. While this is a limitation of the study, we still incorporated the Apple Watch as it also serves the important purpose of demonstrating the potential for commonly used commercial devices to monitor the effect of HRV-directed biofeedback. HRV was only assessed in 1-time domain metric (SDNN) in this study, limiting the evaluation of other HRV metrics with the study outcomes. However, SDNN is 1 of the most common HRV features evaluated when studying resilience or the impact of HRV on psychological or physiological features [19]. Further study evaluating other HRV metrics is needed in the future to determine how other HRV parameters are impacted by short sessions of remote HRV-biofeedback. Another limitation is that we did not have exit surveys to understand why certain individuals were not compliant with study components, such as watching weekly videos or using the HeartMath device consistently. A final limitation is that HRV is not specific and can be impacted by many environmental factors beyond the covariates we controlled in the analysis. This is an important limitation to recognize as there is the potential for unmeasured covariates to impact the results, including such things as ongoing tobacco use and menstrual cycles.

### Conclusion

We demonstrated that fully remote, short HRV biofeedback sessions, using light touch engagement measures, have low compliance rates. However, we did find numerical improvement in psychological assessments over the intervention and follow-up period and alterations of wearable assessed HRV measures in compliant individuals. This supports the need for further evaluation of remotely used short sessions of HRV biofeedback and of the use of wearable devices to monitor response if higher rates of engagement can be achieved.

## Appendix

Multimedia Appendix 1 Study recruitment flyer, screenshot of HeartMath’s Inner Balance smartphone app, and daily circadian pattern of hear rate variability measures.

## Abbreviations

ANS: autonomic nervous system
CD-RISC 10: 10-item Connor-Davidson Resilience Scale
ECG: electrocardiography
HCW: health care worker
HRV: heart rate variability
MESOR: midline-estimating statistic of rhythm
NIH: National Institutes of Health
PHQ-4: Patient Health Questionnaire-4
PPG: photoplethysmography
PROMIS: Patient-Reported Outcomes Measurement Information System
PSS-10: Perceived Stress Scale 10
SDNN: SD of the interbeat interval of normal sinus beats

## Data Availability

The data sets generated or analyzed during this study are available from the corresponding author on reasonable request.
