# Supplementary material for: Remote Short Sessions of Heart Rate Variability Biofeedback Monitored With Wearable Technology: Open-Label Prospective Feasibility Study
Source: JMIR Ment Health. 2024 Apr 25;11:e55552. doi: 10.2196/55552 (PMC11082734; doi:10.2196/55552)

**Figure S1.** A copy of the flyer used for study recruitment.

**
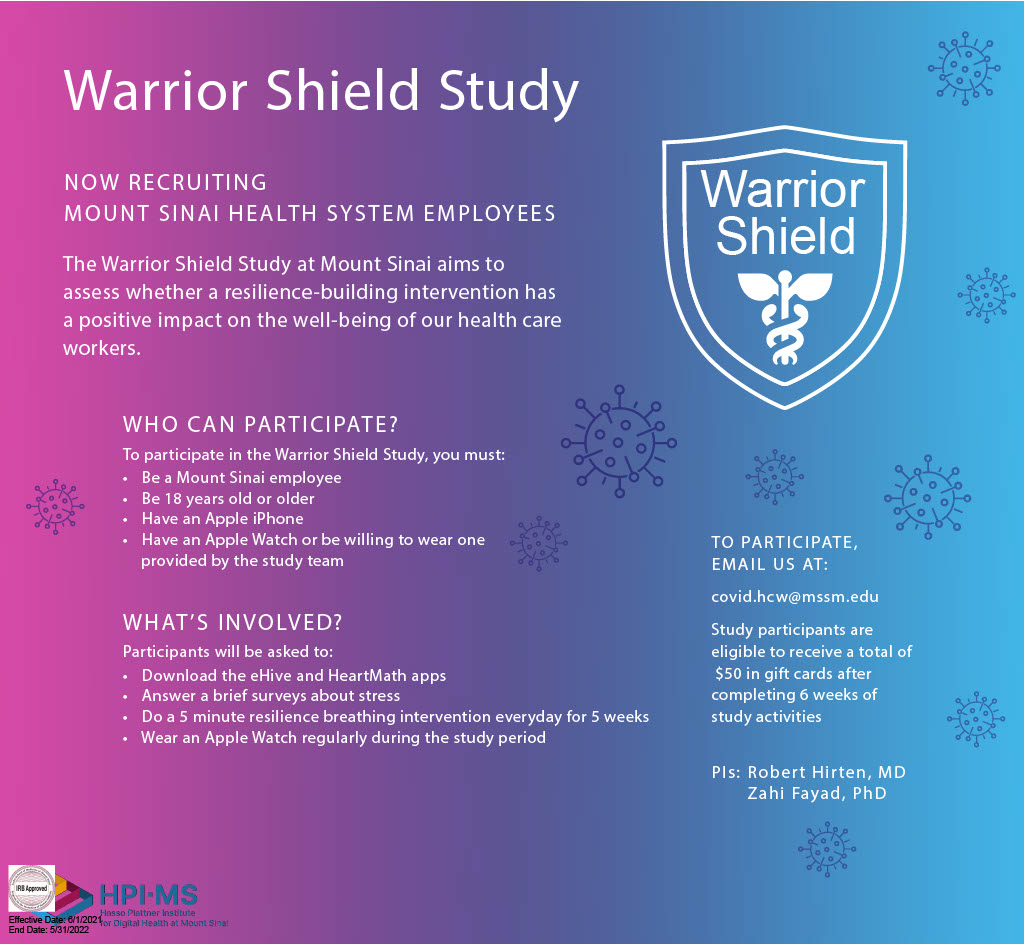
**

**Figure S2.** A screenshot of HeartMath’s Inner Balance smartphone app as seen by a participant. The central flower shaped graphic paces a participant’s breathing. HRV is sensed and visualized during each session in the tracing at the top of the app.

**
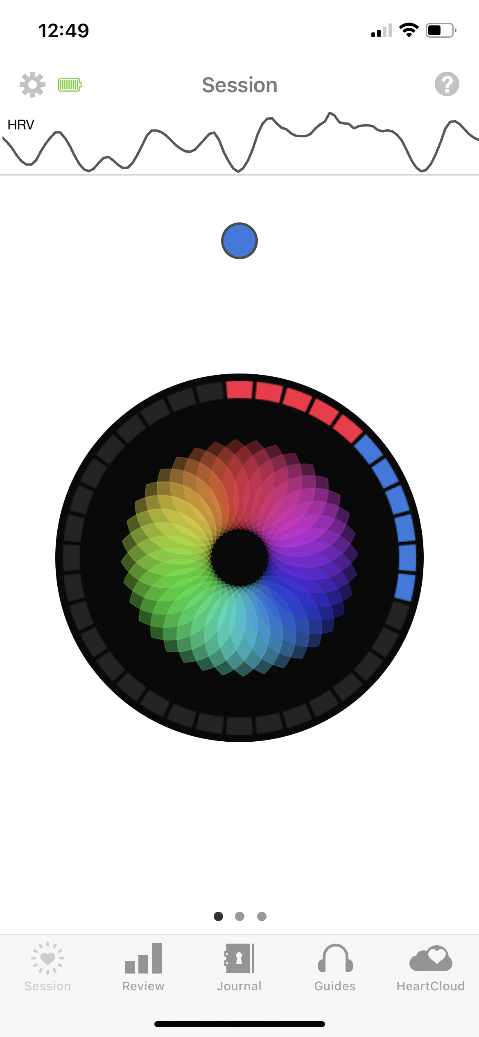
**

**Figure S3.** The daily circadian pattern of HRV measures can be represented by the COSINOR model using 3 parameters. The rhythm-adjusted mean is termed the MESOR. Half the extent of variation within a day is the Amplitude of the curve, and the time of overall high values recurring in each day is the Acrophase.


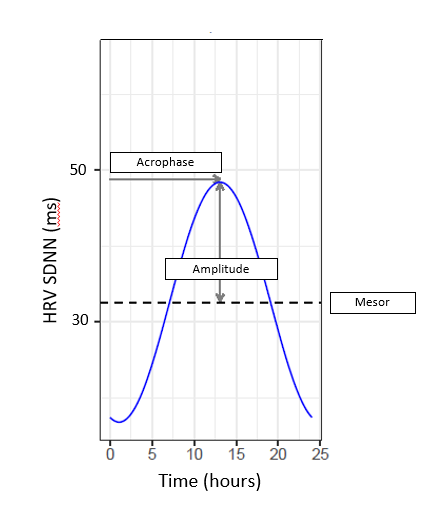

Supplement: Multimedia Appendix 1 [file mental_v11i1e55552_app1.docx]
